# *In-situ* Multimodal Imaging and Spectroscopy of Mg Electrodeposition at Electrode-Electrolyte Interfaces

**DOI:** 10.1038/srep42527

**Published:** 2017-02-10

**Authors:** Yimin A. Wu, Zuwei Yin, Maryam Farmand, Young-Sang Yu, David A. Shapiro, Hong-Gang Liao, Wen-I Liang, Ying-Hao Chu, Haimei Zheng

**Affiliations:** 1 Department of Materials Sciences and Engineering, University of California, Berkeley, California, 94720, USA; 2 Materials Science Division, Lawrence Berkeley National Lab, Berkeley, California, 94720, USA; 3 College of Energy, Xiamen University, Xiamen, 361005, P. R. China; 4 Advanced Light Source, Lawrence Berkeley National Lab, Berkeley, California, 94720, USA; 5 Department of Chemistry, University of Illinois at Chicago, Chicago, Illinois 60607, USA; 6 College of Chemistry and Chemical Engineering, Xiamen University, Xiamen, 361005, P. R. China; 7 Department of Materials Science and Engineering, National Chiao Tung University, Hsinchu, 30010, Taiwan

## Abstract

We report the study of Mg cathodic electrochemical deposition on Ti and Au electrode using a multimodal approach by examining the sample area *in-situ* using liquid cell transmission electron microscopy (TEM), scanning transmission X-ray microscopy (STXM) and X-ray absorption spectroscopy (XAS). Magnesium Aluminum Chloride Complex was synthesized and utilized as electrolyte, where non-reversible features during *in situ* charging-discharging cycles were observed. During charging, a uniform Mg film was deposited on the electrode, which is consistent with the intrinsic non-dendritic nature of Mg deposition in Mg ion batteries. The Mg thin film was not dissolvable during the following discharge process. We found that such Mg thin film is hexacoordinated Mg compounds by *in-situ* STXM and XAS. This study provides insights on the non-reversibility issue and failure mechanism of Mg ion batteries. Also, our method provides a novel generic method to understand the *in situ* battery chemistry without any further sample processing, which can preserve the original nature of battery materials or electrodeposited materials. This multimodal *in situ* imaging and spectroscopy provides many opportunities to attack complex problems that span orders of magnitude in length and time scale, which can be applied to a broad range of the energy storage systems.

Magnesium ion batteries have attracted a lot of recent interest due to its potential for replacing lithium ion batteries for the next generation high capacity batteries^1^. Magnesium ion offers a two-electron charge transfer upon charging/discharging compared to the single electron offered by lithium ion. Magnesium ion batteries have a theoretical gravimetric capacity of 2205 Ah · kg^−1^, which is equivalent to that of lithium ion batteries^2^,^3^. Magnesium has the highest volumetric capacity, 3832 mAh · cm^−3^, among the practical choices of group I and II metals in the periodic table^4^,^5^,^6^. Furthermore, magnesium is a non-toxic, earth abundant and environmentally benign element^2^,^6^. Despite the aforementioned advantages of Mg ion battery, it still faces great challenges in the development of Mg ion batteries, including to find a stable reversible electrolyte with a large electrochemical window, integrate cathode materials with reversible insertion and desertion of Mg^2+^ ions, and overcome the formation of passivating layers on Mg anode^7^.

There has been significant effort in making Mg electrolyte allowing reversible electrochemical deposition/stripping^8^,^9^,^10^. Currently, organometallic Mg ion Grignard based electrolyte is the state-of-art solution which has demonstrated highly reversible Mg electrochemical deposition^3^,^4^. However, organometallic species are highly unstable and easily oxidized^10^, which in turn limit the electrochemical stability window of the solution^11^,^12^. Reversible Mg deposition and stripping from Mg(TFSI)_2_ dissolved in glyme based electrolyte^13^ have proven to be difficult^3^. The toxicity and hazards of Mg(BH_4_)_2_/LiBH_4_ based electrolyte^10^,^12^,^14^ will limit its usage in future Mg ion batteries. Recently, there was a report on an inorganic Mg ion electrolyte. which showed high reversibility and stable electrochemical window near 3 V at room temperature^11^, in which MgCl_2_ and AlCl_3_ salts were dissolved in tetraethylene glycol dimethyl ether (TEGDME) to form Magnesium Aluminum Chloride Complex (MACC) electrolyte. However, the recyclability of Mg electrolyte is still a challenge in general and the mechanisms need further study. Additionally, It is well known that dendritic growth in lithium ion batteries results in battery failure^15^,^16^,^17^ and significant effort has been made to suppress the dendritic growth in lithium ion battery^17^,^18^,^19^. However, it was predicted that Mg electrochemical deposition produced non-dendritic phase due to a higher potential to move Mg active species from the electrolyte bulk salt to the metal surface, i.e., Mg-Mg bond strength is average 0.18 eV higher than Li-Li bond^20^. However, it lacks direct *in situ* evidence of the non-dendritic growth of Mg at the electrode-electrolyte interfaces.

Here, we prepared the MACC electrolyte by following the previously reported procedure^11^ that MgCl_2_ and AlCl_3_ salts were dissolved in TEGDME. After the Mg electrolyte was synthesized (see the methods section), a cyclic voltammetry (CV) was performed inside glove box, which is shown in Fig. 1b. Platinum wire was utilized at the working electrode, while Mg wires were served as both reference electrode and counter electrode. (The details of electrolyte synthesis and *ex-situ* CV measurement are shown in the methods section.) The reductive peak displays a maximum current density near −0.5 V, which is attributed to the deposition of Mg compound^10^. The oxidative peak shows a maximum current density at 0.6 V which corresponds to the electrochemical dissolution of Mg compound at near 0.6 V, as shown in the Supplementary Information (Fig. S1)^10^. Our measurements showed a second oxidative peak around 2 V (Fig. 1b), which was similar to the previously work^10^,^11^. This second oxidative peak was attributed to the electrolyte decomposition and chlorine evolution^21^. No H_2_O reduction or oxidation peak was observed in Fig. 1b, which indicated no obvious H_2_O contamination of the electrolyte. However, when we performed *in situ* studies, electrodeposition was successfully achieved but the deposited layer cannot be stripped. Thus, it suggests an irreversibility issue of the Mg deposition during *in situ* liquid cell TEM experiments.

To develop depth understanding of this *in situ* non-reversibility issue of Mg battery cell electrolyte, we use a novel multimodal approach by a combination of *in-situ* transmission electron microscopy (TEM)^22^,^23^,^24^ scanning transmission X-ray microscopy (STXM) and X ray absorption spectroscopy (XAS)^25^. Although magnesium batteries have been subjected to *in-situ* XAS studies^26^, *in situ* imaging of the electrochemical process in Mg batteries has not been reported so far. The nature of Mg deposition at the electrode-electrolyte interfaces is poorly understood^27^. This is due to the lack of *in situ* observation while reaction proceeds. Barile *et al*. cycled the electrolyte Mg(AlCl_2_EtBu)in THF^27^, then they exposure the electrodeposited materials to water and CDCl_3_ to extract the materials for NMR analysis. It is a rising concern in the field that the battery materials or electrodeposited materials have to be scratch off the electrode by opening battery cell after cycling for characterization. This further processing could introduce possible contamination by chemical treatments or change the nature of the electrodeposited materials during the exposure to air or water during the transfer of materials for characterization. Here, we applied a novel method by using *in-situ* TEM, STXM and XAS to examine the electrodeposited materials in the same electrochemical liquid cell. The electrochemical liquid cell plays an important role as a cross platform for the study of the same sample region without exposure to air. This novel method avoids opening cell or further sample preparation and processing for characterization. Thus, the original nature of battery materials can be preserved, which makes the characterization more meaningful for understanding mechanism. To the best of our knowledge, this is the first time multimodal *in-situ* imaging and spectroscopy of the electrode-electrolyte interfaces in Mg ion batteries. This novel multimodal approach provides the ability to attack complex problems that span orders of magnitude in length and time scale. Our method provides a novel generic method to understand the *in situ* battery chemistry at electrode-electrolyte interface, which could also be applied to a broad range of the energy storage systems, such as Mg ion battery, Na ion battery, Al ion battery, Li ion battery and so on.

## Results

**Figure 1:**
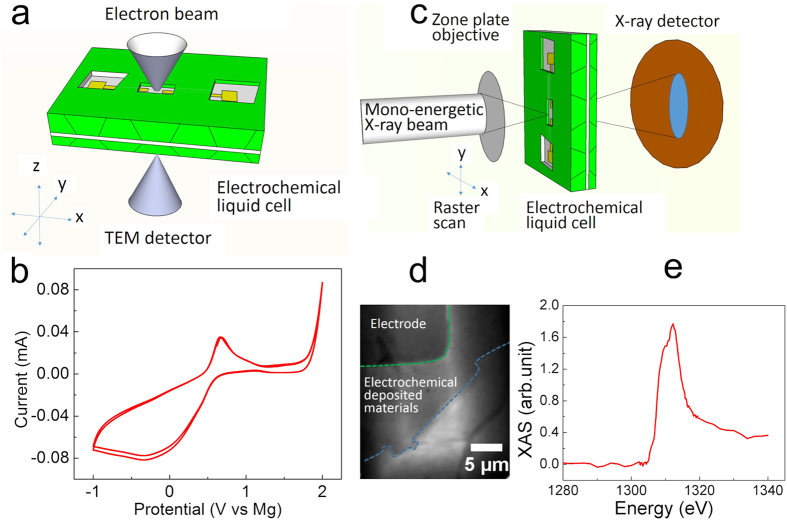
*In situ* multimodal imaging and spectroscopy for energy storage systems. (**a**) A schematic of *in-situ* electrochemical liquid cell transmission electron microscopy. (**b**) *Ex situ* cyclic voltammetry (CV) measurement of 0.267 M MACC 2:1 solution in TEGDME. The working electrode is Pt wire. The counter and reference electrode are both Mg wire. Measurements were obtained at the scan rate of 25 mV · s^−1^ and ambient conditions. (**c**) A schematic of *ex-situ* scanning transmission X-ray microscopy (STXM) of electrochemical liquid cell (**d**) A typical STXM image of electrochemical liquid cell after electrochemical deposition. (**e**) A typical X-ray absorption spectrum of the electrochemical deposited materials on the electrode.

*In-situ* electrochemical liquid cell TEM^28^,^29^ experiments were performed to monitor the electrode-electrolyte interfaces during electrochemical reactions at nanoscale spatial resolution with seconds temporal resolution. A schematic of electrochemical liquid cell is shown in Fig. 1a. Details of the liquid cell fabrication have been reported in previous publications^28^,^29^ and also in the methods section. Mg electrolyte was loaded into one of the reservoirs and the liquid was drawn into the liquid cell by capillary force. Thus, the Mg electrolyte was sandwiched between the two silicon nitride membranes at the window. We sealed cell and connected the bonding wires to the Cu electrical pads on the tip of the sample holder. Then, an electrical bias was applied to the electrodes through the sample holder with a Potentiostat (see methods section). Real time imaging of electrochemical reactions can be achieved under a constant current by *in situ* chronopotentiometry, where movies were recorded using a Gatan Oresis camera with Virtual Dub software. Images were processed using Image J software. After *in-situ* TEM, the electrochemical liquid cell was loaded directly into scanning transmission X-ray microscope (STXM) (Fig. 1c) to examine the electrodeposited materials. A mono-energetic X-ray beam near the Mg K-edge was focused onto the electrodeposited material via the zone plate objective. A typical STXM image and corresponding X-ray absorption from the same sample region is shown in Fig. 1d and 1e with micron scale spatial resolution. The area within green dash line at top-left corner in Fig. 1d was electrode. The area between green dash line and blue dash line are the electrodeposited materials.

**Figure 2:**
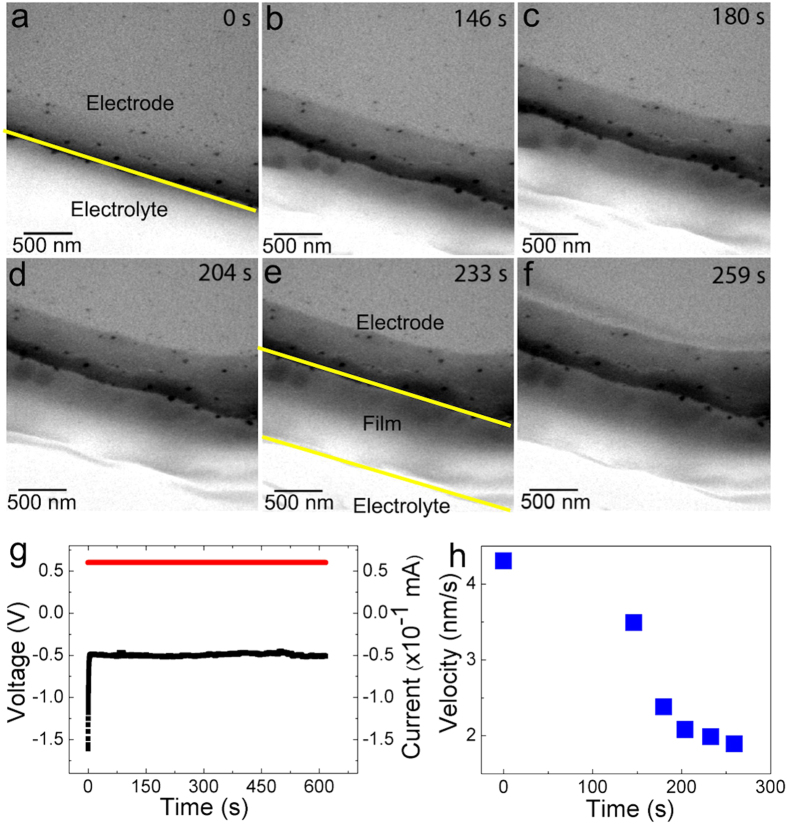
*In-situ* TEM and chronopotentiometry on Ti electrode electrochemical liquid cell. (**a**–**f**) Time evolution of the growth of Mg two dimensional film. (**g**) *In-situ* chronopotentiometry during the cathodic electrochemical deposition. Constant current is shown as red curve. Voltage profile is shown as black curve. (**h**) The forefront propagation velocity of the Mg 2D layer as a function of time.

Figure 2a–f show the sequential images of Mg cathodic electrodeposition process on the Ti electrode when a constant cathodic current of 6 × 10^−2^ mA was applied in an electrochemical liquid cell (see movie S1). A uniform smooth thin film of Mg was achieved and no dendritic growth was observed. Although, other *ex situ* studies indicated non dendrite growth^10^,^30^,^31^, this work provides the first *in situ* study to support the theoretical prediction that Mg batteries should not have safety related issues imposed by dendritic growth^20^.

Before electrochemical deposition, electrode-electrolyte interface was clean (yellow line in Fig. 2a). Some dark spots on the electrode were the topological Ti islands on the Ti electrode (Fig. 2a). Figure 2g shows the *in-situ* chronopotentiometry during the electrochemical deposition of Mg at a constant current of 0.06 mA (red curve in Fig. 2g). The electrical potential (black curve in Fig. 2b) increased from −1.6 V to −0.5 V. During Mg electrodeposition, it maintained at −0.5 V (black curve in Fig. 2g). This indicated the resistance/conductance of Mg film remained the same during charging. This is consistent with previous *ex situ* study^10^. Mg cathodic electrodeposition happens at the range between −1 V to 0 V^10^. The potential of our Mg electrodeposition cathodic process was within this range. During this cathodic process, electrons can travel from Ti electrode through Mg layer to continuously reduce Mg active species during the electrodeposition of Mg layer. The electrodeposited layer was grown between the electrode and electrolyte, indicating by two yellow lines in Fig. 2e. The thickness of Mg film increased continuously (Fig. 2a–f). As a comparison to the solid-electrolyte-interface (SEI) in lithium ion batteries, the internal resistance of lithium ion battery increases when SEI is formed in a charging cycle. However, once the initial SEI layer of Mg ion battery has formed, the inability of electrolyte molecules travelling through the SEI to the active material surface suppressed future SEI growth^32^. The coulombic efficiency of this process in Mg ion battery can be as high as 99.4%, which is higher than the lithium ion battery of 95%^11^. This may explain why Mg film can grow smoothly^20^. The electrodeposited Mg film appeared to be uniform and the contrast of film maintains the same. In Fig. 2h, the propagation velocity of Mg film as a function of time was decreasing, which is probably due to the consumption of electrolyte in the later stage of reaction. However, we did not see the reversible dissolving anodic process when we reversed the bias. Without biasing, electron beam itself cannot trigger electrochemical reaction. Electron beam did not damage the formed thin film under the operating condition.

**Figure 3:**
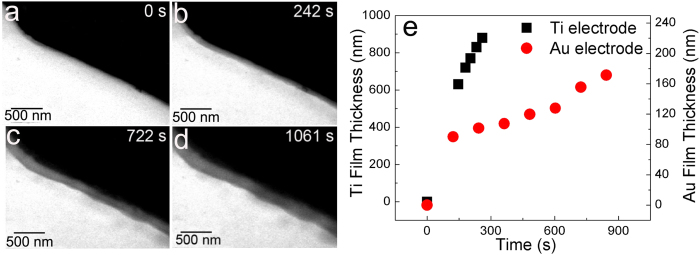
*In-situ* TEM on Au electrode electrochemical liquid cell. (**a**)–(**d**) Sequential images of the growth of Mg film on Au electrode. (**e**) Comparison of growth kinetics of Mg film on Ti and Au electrode. Left y-axis is for Ti electrode. Right axis is for Au electrode.

**Figure 4:**
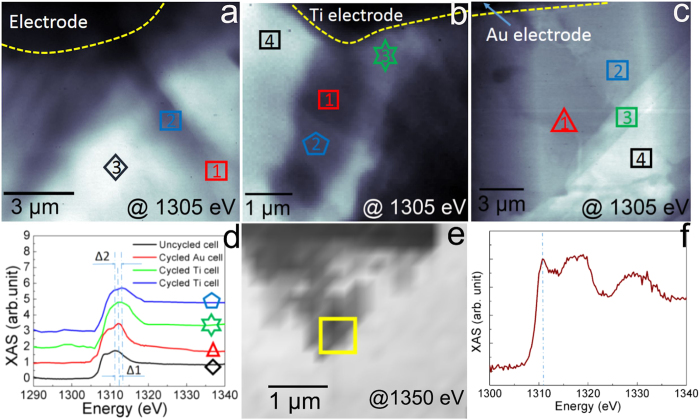
STXM and XAS measurement of Mg deposition in an electrochemical liquid cell. (**a**) STXM image of electrochemical liquid cell filled with electrolyte before charging. (**b**) STXM image of Mg electrochemical deposition on Ti electrode. (**c**) STXM image of Mg electrochemical deposition on Au electrode. (**d**) Mg chemical states of the Mg electrodeposition on both electrodes in the electrochemical liquid cells of (**a**,**b** and **c**). (**e**) Scanning transmission X-ray microcopy of MgO powder. The dark contrast corresponds to the MgO powder. The STXM image was obtained at the photon energy of 1350 eV. (**f**) X ray absorption spectrum of MgO powder in yellow region in (**e**). This spectrum was used to calibrate the instrument.

In order to investigate whether the smooth electrodeposition of Mg is an intrinsic nature or affected by different types of electrode. Another type of electrochemical liquid cell was fabricated by changing Ti electrodes to Au electrodes. It was observed that similar smooth film was deposited on Au electrode. The sequential images of Mg thin film growth on Au electrode are shown in Fig. 3a–d. *In-situ* TEM movie of Mg electrochemical can be found in movie S2 under the *in situ* chronopotentiometry with constant cathodic current of 4.2 × 10^−5^ mA. We observed that the growth rate of Mg thin film on Au electrode was slower than that on Ti electrode, as shown in Fig. 3e. Although the reason for different kinetics on different electrodes still need to be further studied, we can conclude that the non-dendritic Mg electrodeposition is independent of the electrode and likely to be intrinsic. It is noted that no dissolution of electrodeposited Mg thin films was observed either with Ti or Au electrode when the bias was reversed. Unlike Ti metal, which is an inert to Mg metal, Au has possibility to react with Mg to form Mg-Au alloy, according to phase diagram^33^. But the formation of alloy by electrodeposition required the overlap of redox potential regions^34^. We did not satisfy this electrochemical condition. Thus, we did not probe the formation of Au-Mg alloy on the electrodeposited materials by TEM and STXM under this electrochemical conditions. Instead we probed the different coordinated organometallic compound on Ti or Au electrode (Fig. 4). These different coordinated compounds may affect the kinetics of the Mg electrodeposition on Au vs Ti electrode. The kinetics of electrodeposition depends on many factors such as the charge transfer rate, current density, potential dependent cathodic rate constant, bulk concentration of reacting species, and active species diffusion rate^21^. Density function theory (DFT) calculation can play an important role to understand the mechanism of different Mg electrodeposition kinetics on Au vs Ti in the future.

To understand the non-dissolution nature of electrodeposited materials, we further characterized electrochemical species deposited on the electrode using STXM and XAS. To make sure Mg electrochemical deposition can be easily detected under STXM, we continue charged each electrochemical cell for ~40 minutes; a TEM image is provided in Fig. S2. Without opening the liquid cell, electrodeposited materials were not exposed to air and were preserved with their original nature. As a reference material, XAS of MgO powder was measured. Figure 4e shows a STXM image of the reference sample MgO with a corresponding reference XAS spectrum of MgO (Fig. 4f), indicating Mg^2+^ K edge lies at 1311 eV. This is consistent with previous measurement^35^,^36^ and was used to calibrate the instrument. Figure 4a shows a STXM image of liquid cell containing fresh the Mg electrolyte. XAS spectra were collected at three different locations (see Fig. S2 in Supplementary Information). After normalization of each XAS, the maximum absorption peak positions near Mg K-edge at 1311 eV were determined, which was aligned with Mg^2+^. Figure 4b shows a STXM image after charging of the electrochemical liquid cell with Ti electrode from *in-situ* TEM. XAS was performed on four regions in Fig. 4b and the corresponding spectra were shown in Fig. S2 in the Supplementary Information. The regions 1, 2 and 3 of Fig. 4b correspond to the electrochemical deposited material, while the bright area in region 4 in Fig. 4b has much weaker XAS signal corresponding to the remaining liquid electrolyte on Si_3_N_4_ membrane. The electrochemical deposited material on Ti electrode (region 1 and 2 in Fig. 4b) has the maximum absorption at 1313 eV. The solid in region 3 of Fig. 4b with lighter contrast has an absorption peak at 1312 eV (Fig. S2). The similar measurement and analysis were performed after charging Mg electrolyte in an electrochemical liquid cell with Au electrodes from *in-situ* TEM. The absorption peak position of electrochemical deposited materials on Au electrode (regions 1, 2, and 3) is only 1312 eV. Comparing the black line with the blue line and green line in Fig. 4d, we observed two shifts of spectrum Δ_1_ = 1 eV and Δ_2_ = 2 eV from 1311 eV to 1312 eV or 1313 eV after charging Mg electrolyte in electrochemical liquid cell with Ti electrode. This suggests two different configurations on the electrodeposited materials on Ti electrode after charging. Comparing the red line and black line in Fig. 4d, only one blue shift is observed indicating as Δ_1_ = 1 eV in Fig. 4d from 1311 eV to 1312 eV. This suggests one configuration of the electrodeposited materials on Au electrode after charging. It is noted that the electrodeposited materials could be mixture of different configurations, which need further mapping the electrodeposited materials with fine steps.

The electrodeposited materials on both electrodes have a blue shift of ~7 eV (1312 eV for Ti electrode) or 8 eV (1313 eV for Au electrode) from the tabulated absorption edge energy for Mg metal K-edge (1305 eV)^26^. This large blue shift confirms that the electrochemical deposited film is not pure Mg metal. The solid of Mg ion with hexacoordinated structure like MgCl_5_(THF)_1_ has a XAS peak position of 1312 eV^36^, while the hexacoordinated Mg solid like [Mg_2_Cl_3_ · 6THF]^+^ (AlCl_4_)^−^ has a XAS peak position of 1313 eV either from experiments or simulation^9^,^36^. This energy level is the same as our experiment results in Fig. 4d. Also TEGDME is expected to share the same coordination like THF with Mg. This indicates that the electrochemical deposited materials via charging MACC electrolyte in TEGDME result in hexacoordinated organometallic Mg compound. The hexacoordinated Mg compound similar as [Mg_2_Cl_3_ · 6THF]^+^ [AlCl_3_Et]^−^ can exist in both electrodeposited materials and liquid electrolyte and has a XAS peak at 1313.5 eV according to a previous report^36^. After charging on Ti cell, the remaining liquid electrolyte in the region 4 of Fig. 4b has the peak blue shift from 1311 eV (Mg^2+^) (Fig. 4f) to 1313.5 eV (Fig. S2) because the organometallic chemistry happened between Mg^2+^ in electrolyte and solvent during biasing. This is different from the liquid cell with Au electrode. The liquid electrolyte remains at Mg^2+^ (1311 eV) in region 4 of Fig. 4c. Some effort has been made to synthesis and isolate this organometallic Mg compound to identify the structure by using Raman spectroscopy and nuclear magnetic resonance (NMR)^37^,^38^. They found organic donors from the solvent, such as THF, bond to different sites of Mg ion^37^. It is even predicted that if the Mg deposition kinetics is slow enough at the interface, crystal-like precipitates could grow at the electrode-electrolyte interface^36^. Thus, organometallic Mg compounds can be achieved. This coordinated organometallic Mg compounds may need higher overpotential to dissolve^39^, which can be the origin of the non-reversibility of the Mg electrochemical cell. The understanding of this non-reversibility issue can help to develop stable reproducible reversible Mg electrolyte with wide electrochemical windows in future studies.

## Discussion

Although it is still unknown that whether the crystal structure of hexacoordinated organometallic Mg compound on Ti and Au electrode are the same or not, the coordination number of the organometallic Mg compound on Au and Ti electrode are six. Possibly, the hexacoordinated organometallic Mg compound would form crystal with different space symmetry on Au vs Ti electrode even with the same coordination number due to different reaction kinetics. To get crystalline solid-electrolyte interface which may dependent on the some “magic” deposition rate and salt concentration^36^. If got, the crystal structure of electrodeposited materials can be quite different even on the same electrode, which can depend on the thermodynamics and kinetics conditions of the electrodeposition process^34^. It could be single phase or multiphase^34^. Based on the experimental observation, the possible electrochemical process could be summarized as following.


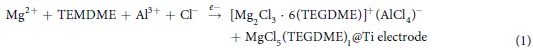



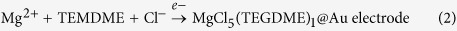


The recent *ex situ* NMR^40^ and X ray diffraction^41^ studies showed the reversible electrodeposited materials are purely Mg dimer^42^ such as [Mg_2_(μ-Cl)_3_(THF)_6_] or [Mg_2_(μ-Cl)_2_(DME)_4_]. However, in our *in situ* studies, we found the formation of Mg monomer MgCl_5_(TEGDME)_1_ on both Ti or Au electrode, which indicated the electrodeposited materials were not purely Mg dimer. The Mg monomer configuration is nucleophilic species which could account for the poor stability^42^. This leads us to believe that the formation of Mg monomer is resulted in the irreversible behavior during the *in situ* studies. It suggests that preventing the formation of Mg monomers or avoiding any impurities facilitating the formation of Mg monomers may help to address the irreversibility issue in Mg batteries.

The electron beam did not damage or affect the electrochemical reaction (Fig. S2). Although we did not observe any water contamination, however, it is worth to note that the purity of commercialized salts in solution may affect the electrochemical performance^43^. In the future, our novel multimodal approach can also help to understand the intercalation of cathode, which is another great challenge^7^ for Mg battery. Active cathode materials such as MoS_2_ and active anode materials such as Mg metal can be deposited onto the Ti electrochemical liquid cell to investigate the Mg intercalation mechanism for Mg battery.

In summary, we have achieved the first *in-situ* multimodal imaging and spectroscopy of Mg electrochemical deposition. Non-dendritic Mg electrochemical deposition was observed. Different from SEI in lithium ion batteries, the Mg film is conducting electrons and there was no voltage drop when Mg film was formed. With X-ray absorption spectroscopy in scanning transmission mode, we found that the electrochemical deposited Mg film is not pure Mg metal but hexacoordinated organometallic Mg compounds with a mixture of monomer and dimer. Therefore, we conclude that the formation of Mg monomers hexacoordinated organometallic Mg compounds has resulted in the irreversible Mg electrodeposition. Our *in situ* TEM study and multimodal characterization shed light on the strategies to avoid irreversibility of Mg ion batteries. This study not only sheds light on designing and improving the performance of Mg ion battery as the next generation batteries for beyond lithium ion battery era but also presents a novel multimodal approach to examine the same sample region with preserved intrinsic nature across the complementary modalities of various microscopy and spectroscopy platforms.

## Methods

### Synthesis of Mg Electrolyte

The synthesis of Mg electrolyte was using a similar methods reporting in the literature^11^. An electrochemically active solution was prepared by adding 0.508 g MgCl_2_ powder (Sigma, 99.99%) and 0.356 g AlCl_3_ (Sigma, 99.999%) with 10 ml of tetraethylene glycol dimethyl ether (Sigma) following the reaction equation 3.


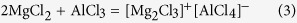


The mixture was sealed with a magnetic bar in a bottle vial to avoid H_2_O and oxygen. The vial was heated in a water bath at the temperature of 60 °C for 9 hours with magnetic stirring on a temperature feedback hot plate in a fume hood. The water bath container was sealed with parafilm to avoid evaporation of the water. The solution was cooling down to room temperature after the heating in the water bath. The resulting solution is light yellow. This clear solution with light yellow color is Magnesium Aluminum Chloride Complex (MACC) Electrolyte. The sealed electrolyte was transferred directly into an argon gas-flow glove box for storage due to its sensitivity to water and air.

A glass vial was sealed with a rubber stopper, while Pt wire (CH Instruments, Inc.) was inserted as the working electrode and Mg wires (Good fellow, 99.9%) were used as the reference and counter electrode. The process was done in a glove box. Mg wire was connected to the Cu wire to make sure that Mg wire was always inside glass to avoid oxidation while the Cu wires act as the current collector for connecting to the external power source. MACC electrolyte was loaded into the glass vial and sealed properly with the rubber stopper in the glove box with Mg wire and Pt wire immersing in the electrolyte. Cyclic voltammetry (CV) was performed using CHI electrochemical station (Model 1100 Series Power Potentiostat/Galvanostat) by connecting Pt wire, Cu wires to the potential station using corresponding clips. The CV was measured with a sweeping voltage from −1V to 2.5 V with a scan rate of 25 mV/s. The *ex situ* CV curve is shown in Fig. 1(b) in the main text. No H_2_O reduction or oxidation peak was observed in Fig. 1b, indicating no H_2_O contamination. Figure S1 shows a reference CV measurement of Mg electrolyte.

### Fabrication of Electrochemical Liquid TEM Cell

The fabrication of electrochemical liquid TEM cells was following our previous work^28^,^29^. The cell contains top and bottom chips. Thin silicon wafers (200 μm, 4-inches, p-doped) was purchased from Virgina Semiconductor (Frederickburg, VA). A 25 nm thick low stress silicon nitride membrane was deposited on the silicon wafer as the viewing window using PVD at the temperature of 850 °C and 140 mTorr. After photolithographic patterning of the wafer, KOH etching creates a suspended Si_3_N_4_ membrane on the Si chip for the viewing window. The dimensions of the windows are 50 μm × 4 μm. Two 90 nm thick Ti or Au electrodes were deposited on the bottom chips with a face to face distance of 20 μm, while 130 nm indium spacer was deposited onto the top chip. The bottom and top chips were aligned and assembled to achieve a liquid cell. It was followed by baking the cell in the oven at 120 °C degree for 1 hour. Then, the wire bonding (westbond) was used to bond Al wires from the Ti or Au electrodes.

### *In-situ* Electrochemical Liquid Cell TEM

As prepared electrolyte was loaded into the liquid cell using syringe, the epoxy was used to seal the reservoir. The liquid electrolyte flowed into the window region by capillary force and was captured between the top and bottom chips as sandwich structures. The electrochemical liquid cell was pocketed into a home-built TEM holder for *in situ* TEM characterization under bias. Both the working and counter electrodes were extended in reservoirs. Aluminum wires were bonded onto each electrode, which can be bonded to the two copper pads on the home-built holder. The holder was inserted into a JEOL 2100 TEM, while connecting to an electrochemical working station (Model 1100 series Potentiostat/Galvanostat, CH Instrument, Inc.). *In situ* electrochemical liquid cell TEM was performed with the accelerating voltage of 200 kV. Real time videos were recorded using Virtual Dub software with a Gatan Orius camera. Liquid cell TEM of Mg electrochemical deposition on Ti electrode under *in situ* chronopotentiometry with constant cathodic current of 6 × 10^−2^ mA (movie S1). Liquid cell TEM of Mg electrochemical deposition on Au electrode under *in situ* chronopotentiometry with constant cathodic current of 4.2 × 10^−5^ mA (movie S2).

### Scanning Transmission X-ray Microscopy and X-ray Absorption

Scanning transmission X-ray microscopy (STXM) and X-ray absorption (XAS) were performed at the bending magnet beamline 5.3.2.1 at the Advanced Light Source (ALS), Lawrence Berkeley National Laboratory, Berkeley, CA, USA. The same electrochemical liquid cell were loaded into the STXM directly after *in situ* TEM measurements without any further sample processing. The STXM and XAS measurements were done utilized a 60 nm outer zone width focusing optics for illumination and processed with a square scan grid of 40 nm or 50 nm steps with a custom developed high frame rate CCD detector. The data were processed using standard methods available in the SHARP-CAMERA software package (found at http://camera.lbl.gov).

## Additional Information

**How to cite this article:** Wu, Y. A. *et al. In-situ* Multimodal Imaging and Spectroscopy of Mg Electrodeposition at Electrode-Electrolyte Interfaces. *Sci. Rep.* **7**, 42527; doi: 10.1038/srep42527 (2017).

**Publisher's note:** Springer Nature remains neutral with regard to jurisdictional claims in published maps and institutional affiliations.

## Supplementary Material

Supplementary Information

Supplementary Movie S1

Supplementary Movie S2
