# Supplementary material for: In-situ Multimodal Imaging and Spectroscopy of Mg Electrodeposition at Electrode-Electrolyte Interfaces
Source: Sci Rep. 2017 Feb 10;7:42527. doi: 10.1038/srep42527 (PMC5301215; doi:10.1038/srep42527)
Supplement: Supplementary Information [file srep42527-s1.pdf]

Supplementary Information

for

In-situ Multimodal Imaging and Spectroscopy of Mg Electrodeposition at Electrode-Electrolyte Interfaces

Yimin A. Wu[1,2†], Zuwei Yin[2,3], Maryam Farmand[4], Young-Sang Yu[4,5], David A. Shapiro[4], Hong-Gang Liao[2,6] ,Wen-I Liang[2,7], Ying-Hao Chu[7], Haimei Zheng[1,2*]

[1]*Department of Materials Sciences and Engineering, University of California, Berkeley, California, 94720, USA*

[2]*Materials Science Division, Lawrence Berkeley National Lab, Berkeley, California, 94720, USA*

[3]*College of Energy, Xiamen University, Xiamen, 361005, P. R .China*

[4]*Advanced Light Source, Lawrence Berkeley National Lab, Berkeley, California, 94720, USA*

[5]*Department of Chemistry, University of Illinois at Chicago, Chicago, Illinois 60607, USA*

[6]*College of Chemistry and Chemical Engineering, Xiamen University, Xiamen, 361005, P. R .China*

[7]*Department of Materials Science and Engineering, National Chiao Tung University, Hsinchu, 30010, Taiwan*

†Present address: Center for Nanoscale Materials, Argonne National Laboratory, 9700 South Cass Avenue, Argonne, Illinois, 60439, USA

Correspondence should be addressed to: hmzheng@lbl.gov

This file includes:

Ex situ cyclic voltammetry of Mg electrolyte Figures S1

Additional STXM images and XAS spectra Figures S2

In-situ Movie Captions S1-S2

■ Ex Situ Cyclic Voltammetry of Mg Electrolyte

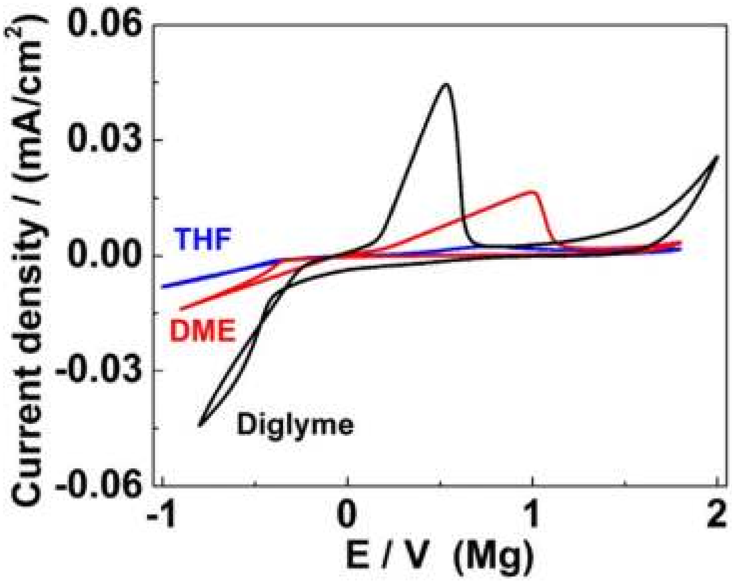

Figure S1. A reference cyclic voltammetry measurement of Mg electrolyte.[1] This reference CV measurement was recorded on a Pt working electrode using Mg ribbon as reference and counter electrode in 0.01 M $Mg(BH_4)_2$ in DGM, DME and THF with a scan rate of 20 mV/s. The overpotential for Mg dissolution in diglyme is the smallest at about 0.5 V, followed by DME (1 V) and THF (1.8 V).

■ Additional STXM Images and XAS Spectra

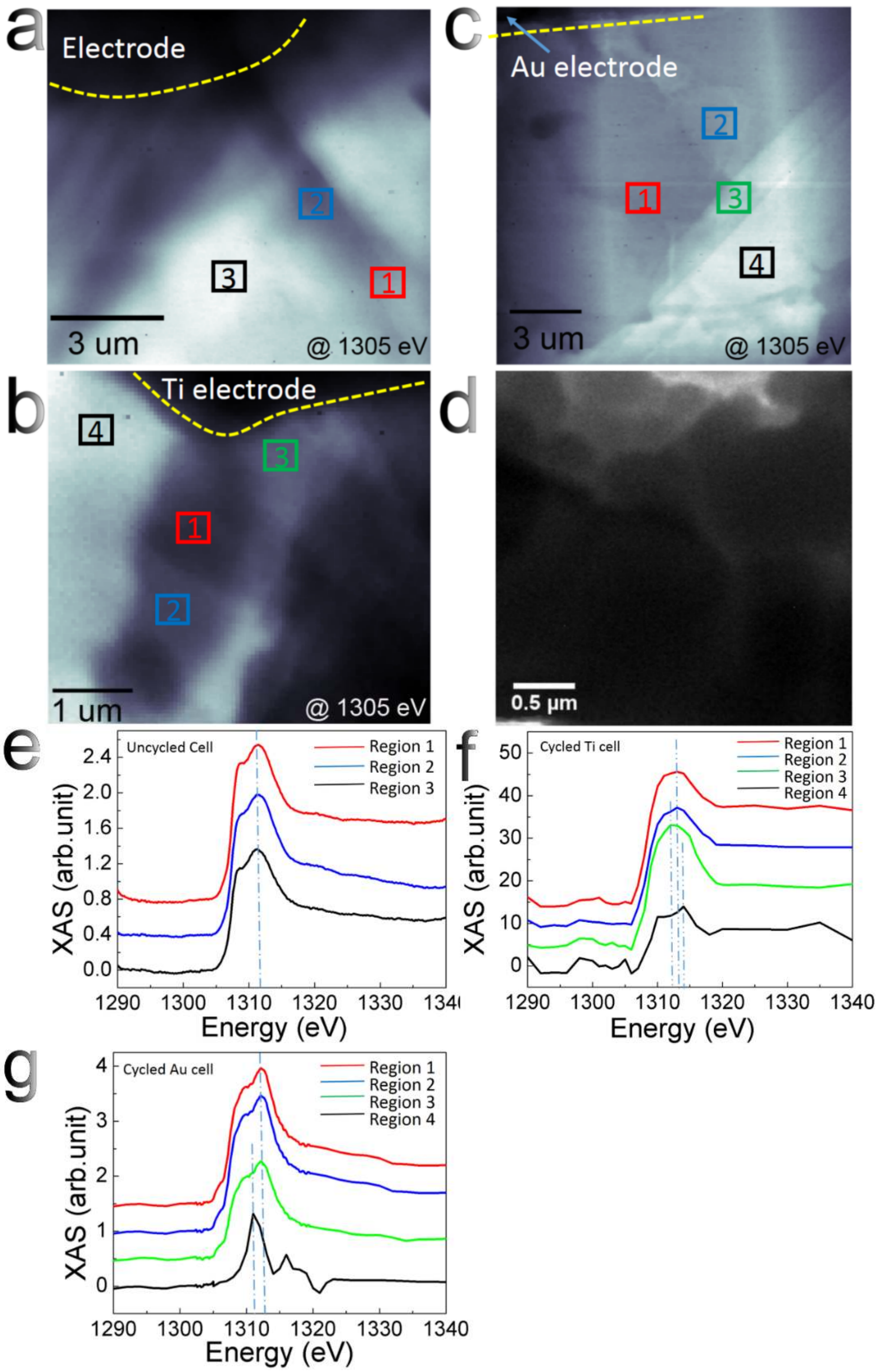

Figure S2. STXM and XAS measurement of the electrodeposited Mg in a liquid cell. (a) STXM image of an electrochemical liquid cell filled with electrolyte before charging. (b) STXM image of the electrochemical liquid cell after Mg electrochemical deposition on Ti electrode. (c) STXM image of the electrochemical liquid cell after Mg electrochemical deposition on Au

electrode. (d) TEM image of electrodeposited Mg materials on Ti electrode after continuous charging of 40min in the liquid cell corresponding to Figure 2 in the main text. The electrode is out of view on the left bottom corner. (e) The corresponding XAS spectra in (a). (f) The corresponding XAS spectra in (b). (g) The corresponding XAS spectra in (c).

■ *In Situ* Movie Captions

Movie S1: In situ liquid cell TEM of Mg electrochemical deposition on Ti electrode under the Chronopotentiometry with constant cathodic current of 6×10^{-2} mA.

Movie S2: In situ liquid cell TEM of Mg electrochemical deposition on Au electrode under the Chronopotentiometry with constant cathodic current of 4.2×10^{-5} mA.

■ References:

1 Y. Shao, T. Liu, G. Li, M. Gu, Z. Nie, M. Engelhard, J. Xiao, D. Lv, C. Wang, J.-G. Zhang and J. Liu, *Sci. Rep.*, 2013, **3**, 3130.
